# Navigating uncertain waters: a critical review of inferring foraging behaviour from location and dive data in pinnipeds

**DOI:** 10.1186/s40462-016-0090-9

**Published:** 2016-10-26

**Authors:** Matt Ian Daniel Carter, Kimberley A. Bennett, Clare B. Embling, Philip J. Hosegood, Debbie J. F. Russell

**Affiliations:** 1 Marine Biology & Ecology Research Centre, School of Marine Science & Engineering, Plymouth University, PL4 8AA Plymouth, UK; 2 School of Science, Engineering & Technology, Abertay University, DD1 1HG Dundee, UK; 3 Centre for Coast and Ocean Science & Engineering, School of Marine Science & Engineering, Plymouth University, PL4 8AA Plymouth, UK; 4 Sea Mammal Research Unit, University of St. Andrews, KY16 8LB St. Andrews, UK; 5 Centre for Research into Ecological and Environmental Modelling, University of St. Andrews, KY16 9LZ St. Andrews, UK

**Keywords:** Movement ecology, Area-restricted search, Satellite telemetry, GPS, Argos, TDR, Animal tracking, Marine mammals, Seals

## Abstract

In the last thirty years, the emergence and progression of biologging technology has led to great advances in marine predator ecology. Large databases of location and dive observations from biologging devices have been compiled for an increasing number of diving predator species (such as pinnipeds, sea turtles, seabirds and cetaceans), enabling complex questions about animal activity budgets and habitat use to be addressed. Central to answering these questions is our ability to correctly identify and quantify the frequency of essential behaviours, such as foraging. Despite technological advances that have increased the quality and resolution of location and dive data, accurately interpreting behaviour from such data remains a challenge, and analytical methods are only beginning to unlock the full potential of existing datasets. This review evaluates both traditional and emerging methods and presents a starting platform of options for future studies of marine predator foraging ecology, particularly from location and two-dimensional (time-depth) dive data. We outline the different devices and data types available, discuss the limitations and advantages of commonly-used analytical techniques, and highlight key areas for future research. We focus our review on pinnipeds - one of the most studied taxa of marine predators - but offer insights that will be applicable to other air-breathing marine predator tracking studies. We highlight that traditionally-used methods for inferring foraging from location and dive data, such as first-passage time and dive shape analysis, have important caveats and limitations depending on the nature of the data and the research question. We suggest that more holistic statistical techniques, such as state-space models, which can synthesise multiple track, dive and environmental metrics whilst simultaneously accounting for measurement error, offer more robust alternatives. Finally, we identify a need for more research to elucidate the role of physical oceanography, device effects, study animal selection, and developmental stages in predator behaviour and data interpretation.

**Electronic supplementary material:**

The online version of this article (doi:10.1186/s40462-016-0090-9) contains supplementary material, which is available to authorized users.

## Background

The need to find food is a fundamental pressure that drives the evolution of animal physiology, behaviour, and life histories [1]. A key question for ecologists is how animals exploit their environment to optimise prey intake and maximise fitness [1]. For air-breathing diving predators, such as marine mammals, sea turtles, and seabirds, foraging poses a unique challenge: within the physiological constraints of breath-hold, individuals must find patchily-distributed prey resources in a three dimensional (3D) dynamic environment [2]. Observing and measuring such behaviour in the field is inherently problematic. However, in recent years, a suite of devices and analytical techniques dedicated to tackling this challenge has emerged [3–8].

Biologging (the “use of miniaturized animal-attached tags for logging and/or relaying data about an animal’s movements, behaviour, physiology and/or environment”; [7]) is changing the way we observe and interpret the behaviour of marine predators [3–6]. Devices allow us to collect an increasing range of data that can be either archived and later retrieved, or autonomously transmitted via acoustic or satellite telemetry, or mobile phone technology (biotelemetry; see [3]). Such data include empirical observations of feeding attempts from fine-scale body movements such as jaw opening [9–11] and lunges measured using accelerometers [12–14], and even physiological measurements of feeding, such as changes in stomach temperature [15–17]. Animal-mounted cameras have complemented such information and contributed substantially to our understanding of how diving predators (both captive and in the wild) search for, capture and handle prey [9, 18–20]. However, datasets from devices such as cameras, jaw magnets, accelerometers and stomach temperature telemetry (STT) loggers are generally limited by small sample sizes and short sampling periods. Moreover, high demands on memory and battery, the need to recover archival tags, or complex attachment procedures limit the use of such devices on wild animals, and thus leave little opportunity for long-term studies with population-level inferences. Nevertheless, direct observations of foraging from these devices can allow us to ground-truth inferences of foraging behaviour made from location and dive (time-depth) data [15, 21–24]. Studies using positional tracking devices and pressure sensors (calculating depth) to measure movement are prevalent, and this type of data has been collected in abundance since the 1980s. Interpreting behaviour from these data, however, can be challenging. A variety of analytical techniques to infer foraging have been advocated, based on assumptions about physiological constraints, behavioural choices and optimal foraging theory (OFT; see Glossary). Most commonly-used approaches have important caveats, depending on the study species and data quality, which we will discuss in detail below.

Many reviews exist of the development, capabilities and applications of biologging devices [3–8, 25, 26]. However, little synthesis has been offered on the data they each collect, which can influence the choice and power of subsequent analysis, and the limitations of commonly-used analytical methods to reliably infer foraging. The purpose of this review, therefore, is to: (i) discuss the range of devices available for tracking horizontal and vertical foraging movements in the marine environment, and the constraints and opportunities presented by the data collected, (ii) discuss the assumptions and relative merits of different approaches to inferring foraging from location and two-dimensional (2D; time-depth) dive data, and (iii) highlight knowledge gaps, providing a point of for future studies. The range of devices and analytical techniques used in foraging studies is extensive across marine vertebrate taxa, especially for seabirds and pinnipeds, for which biologging studies are particularly prevalent [8]. Here, we discuss inference of foraging behaviour in pinnipeds. Although insights may be applicable to other air-breathing marine predator tracking studies, differences in behaviour and device constraints mean that discussion relating to other taxa is outside the scope of this review.

## Devices and data

**Fig. 1:**
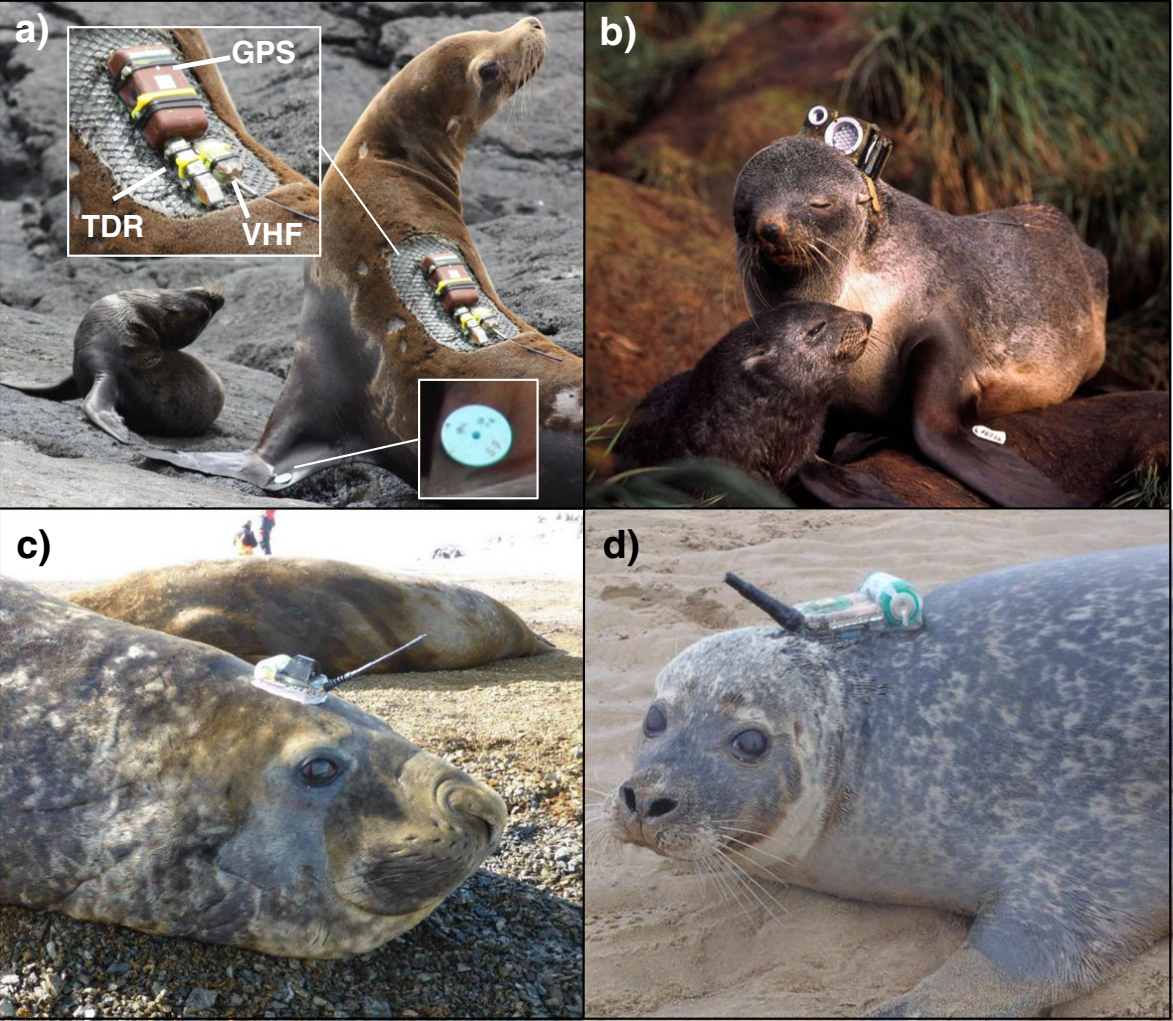
Biologging device deployments. **a** Lactating female Galápagos sea lion (*Zalophus wollebaeki*) with archival GPS and TDR loggers. Archival loggers are favoured for tropical species as Argos satellite coverage is poor near the equator. VHF transmitter aids re-encounter on the colony for device retrieval. Coded mark-recapture tag shown in the fore-flipper (photo: Jana Jeglinski). **b** Lactating female Antarctic fur seal (*Arctocephalus gazella*) with archival video camera (photo: Sascha Hooker). **c** Argos-CTD telemetry tag deployed on a southern elephant seal (*Mirounga leonina*) in West Antarctica. This device records both movement and environmental data simultaneously and transmits the data via polar-orbiting satellites, offering valuable data for ecologists and oceanographers alike (photo: Mike Fedak). **d** GPS-GSM phone telemetry tag deployed on a harbour seal (*Phoca vitulina*) in the North Sea. These devices are a good option for species that frequent coastal waters in less-remote regions (photo: Sea Mammal Research Unit). Note: for scale, devices pictured in (**c**) and (**d**) are roughly the same size

For many years knowledge of pinniped movements was limited to re-sightings of coded mark-recapture flipper tags or brandings [27] (Fig. 1a–b). These observations allow long-term monitoring of survival and dispersal, but offer little insight into where individuals go between hauling-out. Many technological options are now available for tracking animal movement at sea (Table 1; Fig. 1). In this review we focus on devices capable of collecting fine-scale information on foraging movements. Whilst global location sensors (GLS) and smart position or temperature transmitters (SPOT) have been used in foraging studies, they are generally deployed to track migration or broad-scale movement, and foraging inferences are made from behavioural data or higher resolution location data from simultaneously-deployed devices [28–30]. GLS and SPOT tags are therefore excluded from this review.

**Table 1:** Commonly-used tracking devices

Device	Examples	Location Derivation	Data Transmission	Common Applications	Typical Batt. Dur.	Approx. Weight (g)	Advantages	Disadvantages	References
Radio tag (Fig. 2.1a)	Mariner Radar (early studies); Advanced Telemetry Systems MM100 Series	Very High Frequency (VHF) or Ultra High Frequency (UHF)	Acoustic telemetry: radio signal (VHF/UHF)	Early pinniped studies. Short range studies. Relocation for data logger retrieval.	6–12 months	80-200 (early studies); 30	Smaller & lighter than Argos/GPS units. No need to retrieve. Can be used to re-encounter specific individuals on a colony for recovery of archival devices (Fig. 2.1a).	Device must be in line-of-sight range of base station(s) and/or mobile receiver(s) to record locations. Signal can be interrupted by terrain.	[31, 32, 36, 37, 184]
GPS Logger (Fig. 2.1a)	Sirtrack F1G	Fastloc ® GPS	Archival	Mainly individuals with restricted ranges (e.g. lactating female otariids during pup provisioning).	3 weeks – 6 months	215	Fast and accurate location estimates. Lighter than telemetry units. Salt-water switch turns the tag off when the animal dives/hauls out to extend battery life.	Must be recovered to extract data, therefore often needs to be deployed in conjunction with VHF transmitter to facilitate re-encounter on the colony. Study limited to specific timescales (e.g. pre-moult/breeding season).	[36, 37, 185]
Argos relay tags (Fig. 2.1c)	SMRU 9000x SRDL; Wildlife Computers Mk10 SPLASH Tag; Sirtrack KiwiSat 101; Telonics ST-10 PTT	Argos	Argos	Very widely used. Long-ranging pelagic pinnipeds in remote locations.	12 months (depending on power options and duty cycle).	370	Can integrate other sensors such as wet-dry, CTD, or accelerometer. Useful in remote areas where no GSM coverage available. Complete data record can be retrieved if tag recovered. Better coverage in polar regions.	Not all locations & dives transmitted. Data often patchy due to interrupted transmissions. Location estimates can carry high spatial error. Fine-scale reconstruction of movement not possible. Argos coverage poor in areas closer to equator.	[42, 43, 106, 120, 186]
GPS relay tags	SMRU GPS SRDL; Wildlife Computers Mk10 SPLASH Tag	Fastloc ® GPS	Argos	Individuals in remote locations with no GSM coverage or prospect of device retrieval.	3-6 months (depending on power options and duty cycle).	370	As Argos relay tag (above). Solar powered option for extended battery life. Fast and accurate location estimates across most of the globe. Can integrate TDR.	Not all locations & dives transmitted. Data often patchy due to interrupted transmissions. Argos coverage poor in areas closer to equator.	[49, 187]
GPS-GSM (Fig. 2.1d)	SMRU GPS Phone Tag	Fastloc ® GPS	GSM (FTP/SMS)	Pinnipeds in non-remote locations (with GSM coverage).	1–12 months (depending on power options and duty cycle).	370	Many power options including solar panel. All dives and locations can be transmitted. Fast and accurate location estimates across most of the globe.	Individual must enter GSM range in order to transmit data (time lag in data retrieval). Not useful in remote locations. If tag detached at sea before entering GSM range data are lost.	[121, 125, 130, 188]

Battery duration and tag weights are given as a rough indication but are highly dependent on device configuration. References are given to indicate some examples of the application of each device. This table aims to give an overview of commonly-used tagging systems but is in no way exhaustive. Note: most devices, if recovered, can be re-charged, re-programmed and re-deployed. However, due to the low probability of retrieval in many cases, relay devices are generally considered single-use

Pioneering, early pinniped foraging studies used acoustic telemetry such as very high frequency (VHF) radio transmitters to describe at-sea movements [31, 32] and formed the basis of our understanding of pinniped foraging. Feeding was inferred from breaks in the VHF signal from diving, assuming that dives equated to foraging [32], or from dive depth data indicating swimming on or near the sea bed [31]. The scope of this technique was limited by the need for proximity of the animal to an observer or multiple receiver stations in order to triangulate its position [31, 32].

Early time-depth recorders (TDRs) were deployed on several pinniped species in the first diving studies [33–35]. These devices recorded depth readings over time, providing important insight into pinniped diving capabilities. TDRs are archival devices, and have to be retrieved in order to access the data. Archival TDRs and positional loggers (collecting high-resolution Global Positioning System (GPS)-derived location data) are used widely today, but studies are limited to life stages and/or species in which individuals are easily re-encountered and re-captured. For example, many studies use archival devices to track the movements and dive behaviour of lactating otariids (eared seals) [33, 36, 37]. Unlike many phocid (true seal) species, otariid pups have a protracted dependency period, during which they remain on the colony whilst the mother makes repeated foraging trips offshore. As otariid mothers must return to the colony to provision their pups over a longer time period, archival devices can be retrieved with relative confidence. Although some phocid mothers, such as harbour (*Phoca vitulina*) and bearded (*Erignathus barbatus*) seals also make foraging excursions during lactation [38, 39], pups generally spend more time in the water than otariids [40], and may even suckle in the water and move between haul-out sites [41], making the re-capture of a specific individual more challenging. In seminal work, Kooyman [34] studied the dive capabilities of Weddell seals (*Leptonychotes weddellii*) in Antarctica, and translocated them to an area of fast-ice with just one breathing hole, thus ensuring an opportunity to recapture individuals and recover the TDRs. Alternatively, for some species, animals can be re-encountered by predicting the timing and location of their life-history events. For example, Le Boeuf et al. [35] glued archival loggers to the fur of northern elephant seals (*Mirounga angustirostris*) that return to the same colony to moult. Unlike other species in which moulting can be prolonged, elephant seals undergo an annual catastrophic moult, shedding a large quantity of fur at once, during which time they avoid entering the water. Tags are therefore released with the moulted fur on the colony, rather than in the sea, and can be later retrieved. VHF transmitters can be deployed in addition to archival loggers to aid re-encounter of the individual on the colony (Fig. 1a) [36, 37]. For other species, tracking their offshore movements requires a transmitting tag (Fig. 1c–d).

**Fig. 2:**
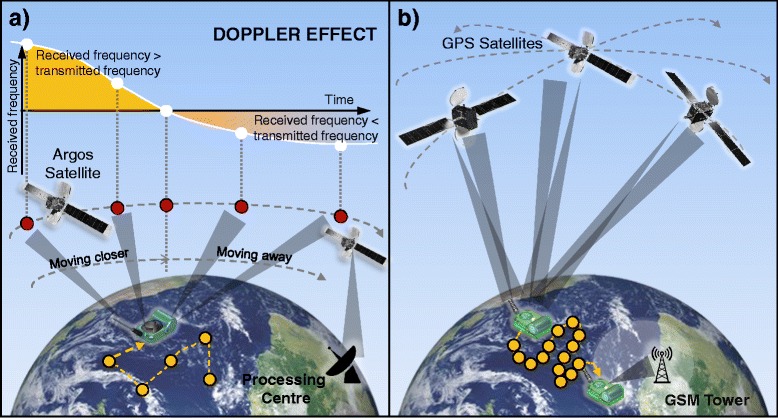
Location detection and transmission methods. **a** Argos satellite tags (adapted from [46]) and **b** GPS-GSM phone tags. Yellow dots represent locations where the tag is at the surface and a location fix is derived. Tag graphics: [60]

Satellite telemetry devices, such as Satellite Relay Data Loggers (SRDLs) were developed in the late 1980s, allowing data to be recorded and transmitted autonomously from anywhere in the world, revolutionising the study of marine predator movements at sea [42–45]. These tags are particularly useful for long-ranging pelagic species, such as southern elephant seals (*Mirounga leonina*; [42]), in which VHF tracking in the open ocean is not possible, and re-encountering individuals for device retrieval is difficult or expensive due to the remoteness of their habitat. These satellite tags were developed to determine location estimates, and transmit data via the Argos satellite system, which calculates the tag’s position using the Doppler-shift in frequencies between the transmitter and low-orbiting polar satellites (Fig. 2a; [46]), relaying the information to a receiver station on land. An important consideration with Argos-derived location data is that location estimates are associated with high uncertainty; the level of which is dependent on how many satellite links are achieved whilst the tag is at the surface (Fig. 2a). Therefore, for species that make long dives with short inter-dive surface durations, such as elephant seals, location quality can be consistently poor [47]. The Argos data-processing system produces location estimates with an associated location class (LC). Poor-quality LCs do not have a measure of spatial uncertainty, and in reality this could range to hundreds of kilometres [46]. As a high-resolution alternative to Argos-derived location data, Fastloc® GPS tags have now been developed, allowing faster location estimation with greater spatial accuracy. Once the antenna is exposed at the surface, it takes less than 100 ms for these devices to collect the data required to estimate a location [47, 48]. Double-tagging individuals with both Argos and Fastloc® GPS technology has allowed more accurate assessment of spatial error and behavioural inferences from Argos data [49–52]. GPS data can be transmitted via the Argos system (Table 1).

With the advent of Argos tags, developers began to incorporate other sensors such as wet-dry and pressure sensors or conductivity-temperature-depth (CTD) sensors alongside accelerometers (measuring tri-axial movement), light intensity meters (detecting bioluminescence in the deep ocean), and fluorometers (to estimate chlorophyll *a* concentration), capable of collecting and transmitting environmental and behavioural covariates simultaneously. In this way, pinnipeds contribute valuable information to both ecological and oceanographic datasets [53–56]. When deployed on long-ranging, deep-diving species such as southern elephant seals (Fig. 1c), these devices can collect environmental data from the entire water column in areas that were previously difficult or expensive to reach (e.g. remote areas of Antarctic water, or areas covered by sea ice; [56]). However, a key limitation of transmission via the Argos system is that data are only transmitted if a satellite is passing overhead while the tag is at the surface, resulting in ‘snapshots’ of location, behavioural and/or environmental data at irregular intervals. For example, Fastloc® GPS tags can record location data at every surfacing, and devices with integrated pressure sensors can record all dives. These data are stored in the device’s buffer memory, however, only a random subset will be successfully transmitted, resulting in patchy datasets [57]. In addition, equatorial regions are likely to receive poorer satellite coverage than polar regions, resulting in sparser data [58]. The impact of these limitations on the analysis of behaviour will depend on the scale of movement of the study species and the specific research question [47, 52].

**Fig. 3:**
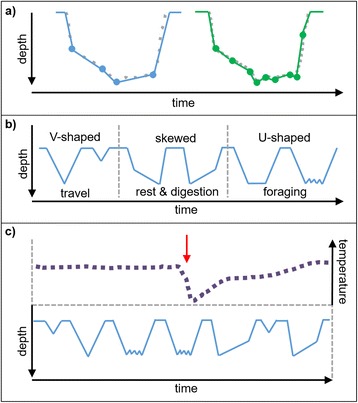
Dive data. **a** Diagram of depth data collected at regular intervals throughout a dive (grey dashed line) and abstracted to inflection points for low resolution (blue dots) and high resolution (green dots) data. This abstraction may be performed using an algorithm on-board the device to reduce the amount of data stored and transmitted. **b** Different 2D dive profiles abstracted from dive data are often used to infer behaviour in seals. **c** Hypothetical example of how stomach temperature telemetry (STT) (top trace) can be used to validate assumptions of foraging inferred from dive profiles (bottom trace). Based on [15], Fig. 1. Arrow denotes feeding event, identified by sharp drop in stomach temperature

A potential solution to the limitations of Argos transmission has emerged in recent years; the option now exists for Fastloc® GPS location data and high-resolution dive data to be archived at-sea and later transmitted via the Global System for Mobile Communications (GSM) phone network (Fig. 1d) [59]. GPS-GSM tags can store data for up to six months in the buffer memory, and as the tagged individual returns to coastal waters (and GSM range) to haul out and rest, the stored data are sent via file transfer protocol (FTP) or text message (SMS) [60] (Fig. 2b). Greater volumes of dive and haul-out data can therefore be obtained, offering better application for fine-scale behavioural studies and recording of rare behaviour [61]. This transmission method also allows higher resolution behavioural covariates to be collected. For example, Argos relay tags and GPS-GSM tags both sample pressure at regular intervals throughout a dive. These data are then abstracted (reduced) to a number of depth inflection points before transmission, from which a 2D depth profile through time can be reconstructed [62, 63]. The number of inflection points per dive varies depending on tag programming: Argos tags typically attempt to transmit four points per dive, whilst GPS-GSM tags transmit many more, giving a much more detailed picture of an individual’s movements underwater (Fig. 3a).

In comparison to Argos, the increased temporal resolution of GPS-GSM technology offers greater potential to recreate spatially accurate animal movements in three dimensions, improving our ability to determine foraging behaviour, and allowing researchers to tackle more complex questions of fine-scale movement and habitat use. Nevertheless, studies in areas without a receiver network must rely on archival loggers or transmission via the Argos system. Despite the higher location accuracy of Fastloc® GPS devices, many researchers still use Argos-derived location data because tags are more economical in terms of battery demand and satellite costs, allowing longer study durations at lower cost [64]. Whilst Argos coverage is poor around the equator, satellite passes are much more frequent towards the poles, making them a good option for polar species [58, 64]. Furthermore, for some species that are wide-ranging, such as southern elephant seals, mapping movements at high frequency and spatial accuracy may be less important in order to successfully identify foraging behaviour.

## Inferring foraging behaviour

Methods of inferring foraging from the data described above generally fall into three categories: (i) use of dive data, (ii) use of location data, and (iii) consideration of movements in three dimensions.

### Inferring foraging behaviour from dive data

In VHF studies during the early 1990s, the presence of diving was used to infer foraging activity in pinnipeds [32]. However, seals may dive for reasons other than searching for prey. For example, individuals may dive for efficient travel [31], and some species also perform resting or digestion dives [31, 35, 65, 66]. Diving datasets collected using pressure sensors can be applied in various ways to infer foraging. From each dive, a number of empirical and geometric measurements can be calculated, including the duration of the dive; duration of bottom time; maximum depth; duration of surface interval; ascent and descent rates; distribution of time allocation across depths (Time Allocation at Depth (TAD) index; see Glossary (Additional file 1)); and 2D dive profile shapes (Fig. 3). The application of each of these metrics depends on the resolution of the data, the temporal scale of analysis, and the specific research question (i.e. whether the study aims to quantify search activity, successful foraging or other aspects of behaviour and physiology).

To quantify foraging in terms of search behaviour at the scale of individual dives, ecologists have proposed that specific 2D dive profile shapes (representing depth over time) can be attributed to different behaviours (i.e. foraging, travelling and resting; Fig. 3b; [35, 67–69]). However, ground-truthing with direct metrics has revealed that the assumed link between dive shape and behaviour may not be consistent between species, age classes or life-history stages [15, 65]. The approach was first applied with northern elephant seals [35], and has subsequently been used for other species [31, 65, 70]. Benthic U-shaped dive profiles are thought to represent either stationary behaviour on, or movement along, the seabed, which have been interpreted as searching for or pursuing prey in benthic-feeding species [15], or resting at depth [31]. The presence of vertical ‘wiggles’ (sinuosity) during the bottom phase of U-shaped dives could also indicate active search behaviour, or pursuit of pelagic prey depending on the proximity to the seabed, and can be used to identify prey capture attempts within dives [28, 71]. However, the ability to detect these movements may be restricted to high-resolution datasets, and determining the proximity of an individual to the seabed is often not possible with Argos-derived location data; accurately matching dive depth to the bathymetric depth of the location where that dive occurred requires a high frequency of accurate location estimates and high-resolution bathymetric data. Skewed shapes may represent drift-dives related to food processing, in which the seal is passively drifting through the water column [35, 72, 73]. V-shaped dives with no bottom time are often taken to represent travelling, or sampling the underwater environment [31, 65]. However, the geometry of a dive is affected by maximum dive depth; dives of similar bottom time may appear as either U or V-shaped depending on the depth. For example, elephant seals forage benthically on deep seamounts [74], and deep foraging dives may appear as V-shaped dives due to the extended time spent in descent and ascent relative to the bottom phase. A dive of similar bottom time at shallower depth with shorter ascent and descent phases would appear as a U-shaped dive.

Direct metrics of feeding have been used to evaluate the accuracy of dive profile shape analysis for identification of foraging. Kuhn et al. [15] used STT loggers to validate assumptions of behaviour from dive profiles for northern elephant seals (Fig. 3c) and found that, although most common on U-shaped wiggle dives (74.2 % of feeding events), feeding occurred on dives of all shapes. Baechler et al. [65] used animal-borne cameras coupled with TDRs, to observe search behaviour in both male and female harbour seals of varying age class. They found that U-shaped dives were a reasonable predictor of search activity for most individuals. However, the accuracy of predicting search behaviour from dive shapes varied for males during the breeding season. Matching video footage with TDR data revealed adult males producing U-shaped dive profiles whilst searching for prey, travelling and roaring underwater (a vocalisation behaviour associated with reproduction) [65]. 2D profiles do not account for lateral displacement underwater; i.e. if an individual remains at constant depth, from a 2D shape we cannot deduce whether they are actively searching or remaining stationary (due to resting, vocalisation, or waiting to ambush prey for example). 3D reconstruction of dives using acoustic positioning arrays, video recorders and accelerometers has revealed that pinniped foraging behaviour during a dive can be remarkably varied and complex [18, 75, 76]. Reconstruction suggests that, if used as the sole analytical technique, 2D profiles may be overly simplistic, introducing a degree of subjectivity to classification of behaviours. Triaxial accelerometers can be particularly helpful to improve our understanding of 3D movement underwater. Head-mounted accelerometers have been used to identify prey-capture attempts in multiple species [12, 71, 77]. However, they can also be used to determine body position and horizontal displacement, and potentially elucidate the particular behaviours associated with individual dives [78]. For example, Sala et. al. [78] deployed TDRs with integrated accelerometers on elephant seals to assess the accuracy of behavioural assumptions from 2D dive shapes. By including data on pitch and roll, the authors were able to visualise the body position of individuals at all phases of dives, and differentiate more effectively between passive drift dives, and active search dives [78]. Moving forwards, combining accelerometer data with dive and location data will increase our ability to infer search behaviour and feeding attempts in 3D. However, accelerometers generate large volumes of data, and the successful transmission of such a quantity of data is currently challenging. Therefore, the deployment of accelerometers is largely restricted to scenarios where they can be recovered. For studies in developed areas, integration of accelerometers into GPS-GSM tags may provide a future solution to this problem.

Whilst U-shaped dives have been used to infer search behaviour at the scale of individual dives, resting dives (with a right or left-skewed shape) may be useful for identifying successful foraging over a broader temporal scale. Drift-rate during rest dives is correlated with an individual’s buoyancy in elephant seals, allowing the inference of body mass gain due to successful foraging [79]. In this way, areas of successful foraging can be mapped [80, 81] and other methods of inferring foraging success from location and dive data can be evaluated [82, 83]. Although similar right and left-skewed dive profiles have been reported in other species [65, 66], comparable relationships between drift-rate and mass gain are yet to be described. This discovery has yielded a relatively simple method of assessing foraging success for long-ranging animals without the need for identifying foraging behaviour. However, phocid seals experience dramatic changes in body mass and composition in response to life-history events, such as fasting (i.e. the post-weaning fast in pups, or due to being hauled out for moulting or reproduction in adults), pupping, and season [84]. Following a period of mass loss, an individual may gain lean mass rather than blubber [85, 86]. Changes in pinniped body composition may also be affected by the lipid content of their prey-type [87, 88], or by seasonal changes in the amount of blubber needed for thermoregulation [89]. In this case, although the individual may be foraging successfully, it is unlikely to experience a positive buoyancy change because lean mass is denser than water [84, 86]. Using drift dives to infer foraging success thus has the potential to overlook successful foraging in some circumstances.

In addition to using dive profiles, ecologists have built theoretical foraging models using dive metrics such as dive frequency, depth, bottom duration, ascent and descent rates, as well as duration of post-dive intervals. Using these models, foraging success is inferred based on assumptions drawn from OFT. Pinnipeds must dive in order to search for food, and the descent and ascent phases of a dive represent the transit to and from a prey patch [90]. OFT dictates that individuals will concentrate their time in areas of successful feeding [1]. Following this, and based on the assumption that foraging occurs during the bottom phase of dives [15, 91, 92], optimal diving theory (ODT) suggests that divers will maximise their time at the bottom phase of a foraging dive [93]. By maximising time spent at foraging depth, and minimising the time spent in transit (descent and ascent) and recovery (post-dive surface interval), individuals increase their chances of prey capture, offsetting the energetic costs of transit between the surface and the prey patch [93]. Importantly, from this perspective, time underwater is maximised over bouts (a succession of foraging dives with minimal surface interval) rather than individual dives [93]. Exceeding the aerobic dive limit (ADL; the point at which lactate begins to build up in the blood [94]) on a single dive, or successive short aerobic dives, will result in anaerobic metabolism, increasing recovery time at the surface and potentially decreasing net energetic gain [2, 95, 96]. Theoretical foraging models based on ODT predict that oxygen will be the limiting factor in dive behaviour [96], and that individuals will consistently dive up to their calculated ADL during foraging dives in order to maximise prey-capture opportunities. Such models therefore predict that foraging success increases with dive duration, bottom time and dive frequency [83].

However, such theoretical foraging models do not account for many of the ecological and physiological complexities that may regulate predator diving. For example, an individual’s physiological capacity to dive to, and remain at depth may vary on a seasonal and diurnal scale [97]. Therefore, predators may adapt their foraging strategies to account for this physiological plasticity. Furthermore, ODT models assume that prey patches are of a uniform density and quality. In reality this is not the case, and depending on the quality and depth of a prey patch, and the level of competition from other predators, maximising time at foraging depth may not always be the most energy-efficient foraging strategy [91, 98]. For example, in an area with a high density of good quality prey patches where prey capture rates are high, the need to stay at depth is less acute; individuals may surface and move on to a new prey patch with lower energetic consequences. The decision to move on may be driven by localised depletion of the food resource, competition, or the need to rest and digest [99]. Likewise, Sparling et. al. [98] have shown that individuals that abandon search and move on early in areas of low prey density maximise net energetic gain. Therefore, in these scenarios, increased bottom time is not correlated with foraging success. Direct observations of feeding attempts have been used to test the performance of ODT models as predictors of foraging success. Such studies have shown that the accuracy of different predictor variables may vary between species, habitats and temporal scale of analysis [21–24, 100]. Viviant et al. [23] deployed accelerometers (measuring jaw-openings as a proxy for prey capture attempts) in conjunction with TDRs on Antarctic fur seals (*Arctocephalus gazella*). They tested a combination of metrics including bottom duration, ascent and descent rates and maximum dive depth as predictors of foraging success. Ground-truthing with accelerometer data revealed that the best predictors varied depending on the temporal scale of analysis, ranging from individual dives to several hours [23]. This suggests that data resolution is likely to be a key factor in the accuracy of different methods of identifying foraging success from dive metrics; the best predictor of foraging success for a particular dive bout may not perform as well when applied across an entire foraging trip. With this in mind, current theoretical foraging models using dive metrics may be too simplistic to accurately describe the dynamics of decision-making in foraging behaviour.

Overall, dive data are a powerful resource when attempting to quantify foraging effort in pinnipeds, in which direct observations of search behaviour or feeding attempts are not available, but should be used with a clear understanding of their limitations. Recent studies suggest that high-resolution dive datasets can be used to inform the best analytical approach for low-resolution data [30, 101]. Moving forwards, tagging a sub-sample of animals with high-resolution devices, cameras or accelerometers where possible may be a good option for future studies in order to identify appropriate analytical techniques.

### Inferring foraging behaviour from location data

**Fig. 4:**
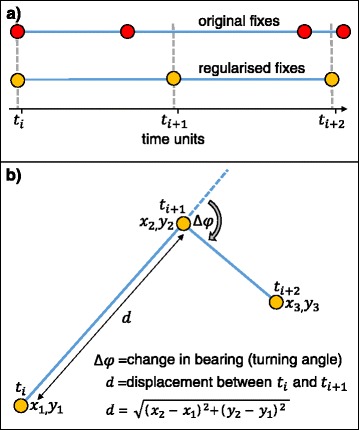
Track metrics. Diagram of successive hypothetical location fixes through (**a**) time and (**b**) space. **a** In order to calculate changes in track metrics through time, it is often necessary to regularise recorded ‘fixes’ (locations) to a constant time step. The resulting regularised fixes are normally connected in space with linear interpolation. **b** Diagram shows two metrics commonly used in movement analyses. Change in bearing (turning angle) is a measure of path sinuosity, whilst the displacement distance between temporally-regularised location fixes can give an estimate of ground speed. By examining changes to these metrics over time different movement patterns can be identified

**Fig. 5:**
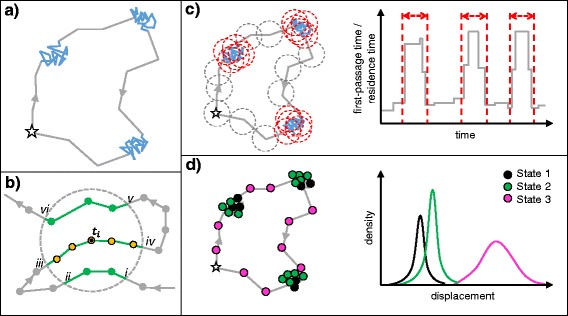
Analytical methods for horizontal movement data. Diagrams show hypothetical track of a central place forager, star represents central place. **a** Two patterns of movement can typically be detected in predator tracks; extensive movements with high displacement and low turning angle (grey lines) and intensive movements with low displacement and high turning angles (blue lines). Intensive movements are commonly taken to represent area-restricted search (ARS) behaviour. **b** Fist-passage time (FPT) is the sum of temporally-regularised location fixes required to leave a circle of given radius in both forward and backward directions from time point *t*_*i*_ (yellow dots). Residence time (RT) includes total time spent in the circle from present (*iii-iv*), previous (*i-ii*) and future (*v-vi*) time steps (green lines), provided that time outside the circle (gap between intersection points *ii-iii* or between *iv-v*) is not above a user-defined threshold. **c** Areas of high FPT / RT can be identified by sliding the circle along the track at each time step. Red dashes denote the areas in space (left) and time (right) taken to represent ARS. **d** Demonstration of a three-state HMM output. Right-hand plot shows posterior Weibull distributions of displacement for three discrete states. Using biological rationale, movement states can be used to infer behaviours (e.g. state 3 with high displacement may be travelling, states 1 and 2 may be either foraging or resting). Presence/absence of diving can be included in the model to distinguish between foraging and resting at the surface [121]

Animal location data can be used to identify and quantify foraging. For central place foragers that make discrete foraging trips to sea, returning to land to rest and digest, or provision young, the duration and extent of these trips are used to make broad observations of foraging effort [102–104]. However, in isolation, trip duration and extent give no information about where individuals are searching for prey, how they are exploiting their environment in order to find it, what proportion of the time at sea is spent foraging in relation to other behaviours such as resting and travelling, and if they are foraging successfully. Within the trip itself, the distribution of time along the track can be analysed, and movement patterns that may relate to specific behaviours can be identified [105]. The simplest way to deconstruct a horizontal track and identify movement patterns is to divide it into segments of straight lines interrupted by turns. In order to quantify the distribution of time along a track, it is often necessary to regularise ‘fixes’ (locations) to a constant time step (Fig. 4a). By interpolating between temporally-regularised locations, the displacement distance and change in bearing between fixes can then be extracted (Fig. 4b). Displacement gives a measure of ground speed, whilst change in bearing (turning angle) can show track sinuosity. Predator movements are often classified into two broad strategies; (1) ‘directed’ travel with little or no meandering, and (2) ‘resident’ behaviour with slower, meandering movement (Fig. 5a) [105, 106]. In the context of OFT, these slower movements are commonly attributed to area-restricted search (ARS) behaviour, indicative of foraging effort within a prey patch [105, 107, 108]. Studies commonly use track metrics to distinguish between directed and resident movement patterns. For example, travel to and from, or between, foraging patches is associated with high displacement between fixes, and small changes in bearing. In contrast, ARS behaviour is characterised by a more sinuous track section with lower displacement [105]. Although opportunistic foraging may occur during directed travel [15, 31, 109], and individuals may search for prey on multiple spatial scales [108, 110], ARS behaviour is often used to quantify when and where predators concentrate foraging effort. Methods of quantifying ARS from track metrics range from simple descriptive approaches (e.g. plotting variable distributions through time and defining a threshold [106]) to sophisticated mechanistic models that can incorporate multiple movement metrics at different data resolutions and account for spatial uncertainty of location estimates [111].

As an alternative to the use of displacement and turning angle, first-passage time (FPT) can be used to distinguish between ARS and directed movement [107]. The FPT is defined as the time taken for an individual to cross a virtual circle of given radius [107]. The circle is centred on the location fix, and the FPT is calculated by summing the number of temporally-regularised time steps taken to leave the circle boundary in both forward and backward directions (Fig. 5b). The areas in which individuals concentrate their time can be identified by sliding this circle along the track (Fig. 5c). FPT analysis is conceptually simple and relatively straightforward to implement. Nevertheless, certain limitations restrict its accuracy for pinnipeds. For example, Fauchald and Tveraa [107] proposed that the chosen radius of the circle should be equal to the diameter of a typical prey patch. This, however, assumes that all prey patches will be circular, and of equal density, and that predators are repelled by patch boundaries. In reality, the density and distribution of pinniped prey resources is rarely known, and may vary along a single track. Seals may access multiple habitats, and target different prey types during a foraging trip, with subsequent effects on foraging behaviour and the scale of search [108, 112]. The circle radius chosen for FPT analysis will therefore be somewhat arbitrary, and defining one scale for the entire track may be inappropriate [108].

‘Residence time’ (RT) analysis was developed as an adaptation of the FPT concept to attempt to overcome the aforementioned limitation. It can take account of previous time spent in the same circle (Fig. 5b) [108]. This means that the analysis is more effective at identifying foraging areas in heterogeneous environments, as it gives a cumulative measure of habitat use [108]. Furthermore, RT analysis uses a statistical approach (penalised contrast algorithm; [113]) to identify ARS, rather than relying on visual inspection of model output [108]. Barraquand and Benhamou [108] show that the RT approach is also less influenced by data resolution than FPT analysis. Whilst this method represents a step forward from FPT analysis, it still relies on the user to define certain parameters (i.e. the amount of time an animal is out of the circle before that path segment is discounted; Fig. 5b). Furthermore, as with FPT, RT analysis cannot account for the uncertainty related to location estimates, or give a measure of uncertainty related to assumptions of foraging behaviour. In recent years, sophisticated modelling techniques have been developed that can perform these functions in a more objective manner [111].

State-space models (SSMs) have multiple applications for animal movement studies [111, 114]. SSMs can be used to improve the spatial accuracy of location estimates, and/or estimate movement modes [115]. Location estimates can be processed with an SSM (e.g. Kalman filter; KF) to reduce spatial errors [116]. The SSM predicts the current state (location) together with its associated spatial error. For data collected since 2008, Argos have offered the option to receive location estimates derived by a KF, rather than the original least squares (LS) algorithm [46]. Once a prediction for a location is made, the algorithm updates all predictions of previous locations using a weighted average, giving more weight to estimates with higher certainty. Recent studies have tested the accuracy of both KF-derived and LS-derived Argos locations for phocids using simultaneously collected high-resolution Fastloc® GPS data as a measure of ‘true’ location [50, 51]. Silva et al. [51] found that 82 % of KF-derived harbour seal locations were within 5 km of the ‘true’ GPS positions, compared with 73 % of LS-derived locations. By improving location accuracy, SSMs can increase the potential of low resolution telemetry datasets for investigation of behaviours [50, 51].

SSMs have been developed that can estimate movement modes from location data. The cleaning of the location data, to improve accuracy, can be combined with state estimation [115, 117]. Alternatively, data are used directly for state estimation within a hidden Markov model (HMM); such data must be spatially accurate (GPS-derived), or already cleaned using an SSM to improve accuracy [111]. HMMs are SSMs in which a finite number of discrete states are defined. States are estimated based on the distribution of movement metrics through time [118]. HMMs take input movement metrics, such as displacement and consistency in travel direction, and generate unique posterior distributions for each, based on a specified or estimated number of behavioural states (Fig. 5d). A recently-developed R package “moveHMM” makes building ML HMMs accessible for researchers outside the field of statistics [119].

SSMs represent a powerful tool for foraging analysis in that they can combine multiple movement metrics from tracking data and estimate movement states with a higher degree of statistical robustness than other commonly-used methods [111, 117, 120]. For example, unlike FPT and RT approaches, SSMs can distinguish between multiple movement patterns without needing to apply thresholds to movement metrics or analytical scale [117]. Using biological rationale, ecologists infer behaviours from the discrete movement states identified by the SSM. SSMs enable us to quantify foraging behaviour in relation to other behaviours such as resting and travelling, and thus tackle more complex questions of how these activities interact through time and space [117, 121–123]. Importantly, models can test the influence of explanatory covariates on the probability of switching into a certain movement state. For example, Morales et al. [124] demonstrated how SSMs can be used to investigate the influence of environmental factors on foraging decisions. Furthermore, models can be applied in either a frequentist Maximum Likelihood (ML) or Bayesian framework. Although ML models are more tractable and computationally less costly, they typically require data to be in uniform time steps with little associated error in order to make the calculation [118]. Bayesian models do not have such constraints, and therefore can account for spatial uncertainty and irregular time series arising from coarse tracking data [114]. This makes them a good option for data collected via the Argos system. However, processing time for Bayesian models is greatly increased due to the associated computational demand.

Pinniped location data has been an important resource for developing and testing the utility of SSMs for animal movement studies [117]. SSMs have now been applied to large datasets to investigate diverse questions including intrinsic and extrinsic drivers of variation in foraging behaviour [121, 122]. However, despite the relative advantages of SSM approaches over other commonly-used analytical methods, they share a common limitation if only applied to location data. Using only horizontal movements, these techniques assume that individuals make discrete journeys in order to forage, but return to the central place to rest. This paradigm therefore assumes only two behavioural modes at sea; travelling and foraging, and attributes all slow, sinuous movements to ARS behaviour [117, 122]. Given that many pinniped species rest at sea during foraging trips either at the surface [31, 105, 121, 125], or beneath it [66, 72], producing similar movement patterns to ARS, using location data in this way may over-estimate foraging behaviour. In order to overcome these limitations it is necessary to consider movement in three dimensions.

### Combining dive and location data to improve foraging models

Recently, Russell et al. [121] used an SSM to investigate the possible drivers of contrasting population trajectories for sympatric grey (*Halichoerus grypus*) and harbour seals in the North Sea. They combined location data with simultaneously-collected dive data. Using consistency in travel direction and displacement distance between temporally-regularised location fixes, the model identified between high transit rate with small changes in direction, and slower, more sinuous movement [121]. However, incorporating presence/absence of diving in the model allowed the authors to infer two behavioural states from slow movements; ‘foraging’, and ‘resting at surface’ [121]. Moreover, where previous studies had excluded data within proximity of the coast to avoid classifying time spent hauled out on land as foraging, using the dive data, they were able to include resting on land as a further behaviour in their analysis of activity budgets [121]. In this way, the analysis was able to capture coastal foraging that may have been excluded by applying a coastal buffer [121]. Importantly, the study found that >10 % of the seals’ activity was attributed to resting at the surface whilst at sea, highlighting the importance of combining track metrics with dive data to ensure that resting behaviour is not mistakenly classified as foraging [121]. For species that commonly rest underwater, however, such as elephant seals, incorporation of presence/absence of diving would not be a satisfactory method of distinguishing between resting and foraging. In this case, models could attempt to distinguish resting dives by their shape, duration or vertical displacement rate compared to foraging or travelling dives, and thus inform behavioural states in the same way.

Although either dive or location data may be used in isolation to identify foraging with traditional methods or SSMs, the accuracy of analysis is often scale-dependent and highly influenced by data resolution. Furthermore, using one of these data types alone may over-simplify at-sea behaviours, leading to over or under-estimation of foraging activity. Including both dive and location metrics in analytical models lends more information, and therefore more power to foraging analysis [126]. Bestley et al. [120] incorporated dive depth and duration, as well as post-dive surface interval into an SSM with horizontal track metrics to describe foraging for multiple Antarctic pinniped species. The use of vertical data improved the capacity of the model to identify where foraging bouts occurred [120]. Increasing the accuracy of foraging models in this way will allow ecologists to identify important foraging habitat with greater certainty, and improve the effectiveness of conservation management. Moving forwards, SSMs represent a powerful tool for tackling complex questions of both the spatial and energetic dynamics of foraging. Furthermore, the ability to incorporate environmental covariates in SSMs may prove vital in unravelling how oceanographic processes drive spatial and temporal patterns of foraging behaviour [114, 127, 128]. SSMs have great potential for maximising the utility of tracking datasets, and the combined advantages they offer cannot currently be equalled by any other approach that we know of. SSM techniques not only allow us to identify foraging behaviour in a more statistically robust manner than traditional methods, but they allow us to do so by combining multiple data types (e.g. dive and location data) and qualities (e.g. Argos and GPS data) in the same analysis, thus maximising the application of available data resources [61, 121]. However, a trade-off exists between the computational tractability and simplicity of models, and biological realism. For example, combining multiple data types will improve the biological realism of inferred behavioural states, but will increase computational demand and technical complexity. Nevertheless, SSMs for animal tracking data continue to be refined and developed, and these models represent our best option for improving our understanding of pinniped foraging dynamics as multi-year tracking datasets grow in abundance. This progress will depend, however, on ecologists collaborating closely with statisticians, sharing their code openly alongside published studies.

## Future directions

### Data resolution

**Fig. 6:**
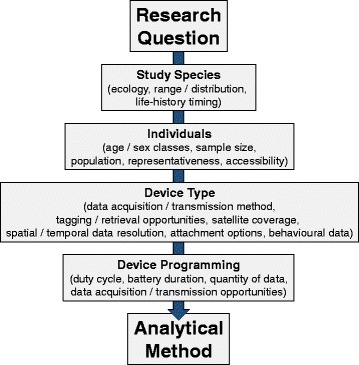
Choosing the right analytical method. Choosing the appropriate analytical method will depend upon careful consideration of some key aspects of the study. Key aspects are given in bold, subsequent considerations are shown in parentheses

Foraging can be classified at the scale of surface movement patterns, individual dives, or even parts of dives. With all attempts to infer foraging from tracking data there are important considerations to be made. Firstly, models should ideally detect foraging on the scale of search of the individual. For animals with small ranges of movement, detecting search behaviour may require data at high levels of spatial and temporal resolution. The scale of movement may therefore be too fine to detect with data transmitted via Argos, and researchers may find that model parameters are defined by the data resolution rather than the biology [64, 83, 110]. This may result in under/over-estimation of foraging. For example, a recent study used SSMs to compare activity budgets for grey and harbour seals [121]. The study combined Argos SRDL data with GPS-GSM data. In order to utilise both data types, SSMs were fit to assign movement states to 6 h intervals. Whilst the resolution was suitable to quantify activity budgets for grey seals, determining between travel and foraging proved problematic for harbour seals, probably because they typically forage closer to shore, and thus do not exhibit long periods of travelling [121]. The SSM models for harbour seals performed better when GPS data were used on a 2 h resolution interval [129]. Secondly, the research question will also dictate the resolution of data required; in order to investigate the fine-scale movements of harbour seals within an offshore windfarm, Russell et. al. [130] used an SSM with fifteen minute intervals. In this case, with a 2 h interval, it would not be possible to determine if individuals trace specific structures, or to distinguish between foraging and travelling around and between these structures. For long-ranging species moving across ocean basins, in which behaviour may switch between migration and residency, a small number of location fixes per day may be enough to detect discrete behaviours. Therefore, when designing tagging studies, researchers should be mindful of the spatial and temporal data resolution required to accurately identify changes in movement patterns for their study species and research question, and choose a device and sampling rate that will capture this signal (Fig. 6; [64]). Nevertheless, increasing duty cycles will likely have a negative effect on the duration of the battery, and so, the trade-off between sampling frequency and duration needs to be carefully considered.

Biologging device deployments are normally costly and logistically demanding. With all devices, the quantity and quality of the data transmitted will depend on the battery life, transmission opportunities, duty cycle, satellite coverage and animal behaviour [58]. However, Patterson and Hartmann [58] pointed out that researchers often rely on trial and error to optimize sampling regimes, resulting in unhelpful datasets. They suggest that pooling datasets across species and regions to compare tag performance could help in designing optimal data collection strategies. Moreover, they showed that synchronizing transmission attempts with satellite passes can improve data throughput and battery performance [58]. Studies of this technical nature are extremely helpful, but have received little acknowledgment in subsequent published studies. We suggest that such theoretical research should be consulted before selecting and programming devices to avoid incomplete datasets and to maximise the utility of the data. Furthermore, improving biologging data utility will depend upon ecologists collaborating with technicians and engineers to improve device battery performance and maximise data capture.

### Device effects

Although technological advances are allowing us to minimise the size and weight of biologging devices, there is substantial evidence to suggest that some methods of handling animals for tag application, and the physical effects of the tag itself, may alter the subsequent behaviour of the individual, and perhaps its prey [131–134]. For example, head-mounted cameras with strobe flashes have been reported to affect the diving behaviour of their pinniped carriers, and/or the prey on which they forage [131]. In contrast to flying seabirds, where device weight can have a large effect on the energetics of flight [135], drag caused by tag placement is a greater concern in pinnipeds [136]. Hazekamp et al. [132] showed that externally-attached devices such as SRDLs can change an animal’s hydrodynamics and potentially alter their physiology and behaviour. Tag designers face a challenge in that a device’s antenna must break the surface in order to receive a location estimate and/or transmit data. This often requires tag placement on the head, neck or back. Attaching tags in a caudal position would likely reduce device-induced turbulence [137], however this would compromise data collection and transmission. Whilst the effects on animals will only last as long as the device is attached, ecologists should be mindful that the movement patterns observed in their data may carry some bias. In addition to these concerns, this also raises ethical considerations about the welfare of the individual, particularly for juveniles and smaller species, in which drag effects may be more severe [138]. As we continue to rely on biologging data to inform the conservation management of species, more research is urgently needed in this field in order to assess the potential bias in existing datasets, refine capture and tag application methods, and improve the hydrodynamic footprint of externally-attached devices.

### Considering the environment

The vast majority of marine predator studies that recreate animal movements from tracking data do so in geographical space; i.e. they assume that the individual is moving through a still medium with no physical forces acting upon them. The reality is that ocean tides and currents can have a strong and dynamic influence on movement and therefore how we infer behaviour [139]. Gaspar et al. [139] reconstructed the movements of a migrating leatherback turtle (*Dermochelys coriacea*) in both geographical and hydrographical space (accounting for ocean currents). They showed that currents can have a large influence on how we interpret track tortuosity, and therefore identify ARS. The study demonstrated how overlooking ocean currents can compromise our ability to successfully identify foraging activity, particularly in areas of high turbulence which are normally associated with high prey density and productivity [139]. Moving forwards, it is vital that researchers consider the dynamic physical nature of the individual’s environment before attempting to interpret behaviour from location data alone. For example, an individual foraging on pelagic prey in the water column may be moving with the current, whilst a benthic-foraging animal may be attempting to remain in one place, actively swimming against the current. This has important implications for the way data are interpreted and how researchers assign behaviours to observed patterns. In the latter scenario, if we do not consider currents, an individual may appear to be stationary or resting underwater when in fact it is foraging, and perhaps expending significant energy in maintaining position. One way to avoid this error is to exclude data in areas of high tidal flow [121]. It is not understood, however, exactly how predators exploit ocean currents and this approach may fail to identify potentially important foraging habitat [140–144]. Therefore, for foraging studies, other approaches that capture the influence of currents on the movement of the instrumented animal should be explored. For example, a drift covariate may be incorporated in hierarchical models of animal movement to account for ocean currents [145]. We suggest that studies similar to that of Gaspar et al. [139] should be conducted with multiple pinniped species in varied oceanographic conditions to assess the effect of currents on detection of ARS for commonly-used methods. Deploying STT devices or accelerometers in conjunction with tracking devices may help to inform researchers about how their study species exploit ocean currents during foraging [146].

Considering the environment in which the study species exists is important not only for the accurate identification of foraging, but also for understanding how abiotic (i.e. oceanographic) covariates may be driving observed behaviour [114, 128]. Bailleul et al. [80] used data from animal-borne CTD sensors in conjunction with drift-dive analysis to determine the unique oceanographic features of important foraging zones for southern elephant seals. Studies such as this may provide key information to aid conservation managers and marine spatial planners in designing effective protection for marine predators. A major advantage of environmental sensors deployed on free-ranging marine predators is that they provide valuable information for ecologists and oceanographers alike [53–55]. In addition to data from animal-borne sensors, a comprehensive suite of remotely-sensed, and buoy-recorded physical oceanographic data is available to give a more complete picture of oceanographic processes, (for example from the Physical Oceanography Distributed Active Archive Center (PODAAC), the British Oceanographic Data Centre (BODC), the National Oceanographic and Atmospheric Administration (NOAA), and the NERC Earth Observation Data Acquisition and Analysis Service (NEODAAS)). Data on sea surface temperature (SST), bathymetry, tidal vectors, sea-ice coverage and wind shear stress, used in conjunction with tracking datasets, are now allowing ecologists to build a greater understanding of how populations may respond to climate anomalies [147], and potentially exploit dynamic oceanographic features, [30, 112, 146, 148–150]. Moreover, incorporating environmental covariates in SSMs may allow us to simultaneously improve our ability to identify foraging behaviour and determine habitat preference, whilst taking into account the uncertainty in locations and assumptions about classifying foraging [114, 124, 128]. Moving forwards, combining data sources to improve our ability to identify and predict behaviours from marine species in this way could inform novel conservation approaches such as Dynamic Ocean Management; “management that rapidly changes in space and time in response to changes in the ocean and its users through the integration of near real-time biological, oceanographic, social and/or economic data”; [151].

### Population-level inferences

Although we are drawing an increasingly detailed picture of marine predator foraging behaviour, research has tended to be heavily focussed on a handful of species and demographic classes [26, 152]. In pinniped tracking studies, there is a general bias towards reproductive females [152], as many species are tied to land throughout the pupping and provisioning phase of the breeding cycle, and are thus easier to catch for tag application and retrieval. This is most notable in the otariid literature. Nevertheless, foraging strategies are known to vary seasonally, between the sexes [104, 122, 153–155], age classes [123, 156–158], and indeed between individuals in general [92, 112, 159]. Due to cost and logistics, tagging studies are often constrained by relatively small sample sizes. However, in order to answer research questions that will have some benefit to the conservation management of species, it is often necessary to make population-level inferences about foraging and habitat use [160]. Fully understanding population dynamics and potential threats may therefore depend on examining the behaviour of individuals from across their range, sexes and age classes [160]. A further consideration is that the individuals selected for a tagging study may not always be representative of the wider population. Logistical constraints mean that tagged animals are rarely selected at random. For example, it may be necessary to select individuals from the periphery of a colony in order to minimise disturbance, or known animals may be preferentially selected based on their contribution to long-term datasets, or robustness to handling. However, it is not known how the capture or selection method may introduce bias to population-level inferences of behaviour; i.e. animals taken from the periphery of a colony may be in poorer condition, which may be reflected in their behaviour at sea. Moving forwards, when investigating population-level foraging, researchers should consider the number of tags that need to be deployed, and whether they can logistically obtain a balanced and representative sample (Fig. 6). For a more detailed discussion on representativeness of study sample in population-level tracking studies, see [160].

Animal movement studies face the inherent challenge that the quantity of data may be disproportionate between individuals or groups [160]. Furthermore, location and dive observations are autocorrelated, and the use of multiple observations per individual is considered pseudo-replication [161]. Whilst detailed discussion of this is beyond the scope of this review, they are important considerations when analysing such datasets, and researchers should select the most robust statistical tools available to them. In recent years, mixed effects models and generalized estimating equations with correlation structures have become more prevalent in pinniped studies, and can help to overcome these challenges. For more discussion on this, see [160–162].

To discover how individual differences in foraging strategies arise, we must focus more research attention on ontogeny [144]. First-year survival in pinnipeds is naturally low, and variable between years, and has an important effect on population dynamics [163–166]. However, a dearth of information exists on the factors that affect the development of successful foraging behaviour [26]. In order to address this knowledge gap, researchers should attempt to track recently-weaned pups as they explore their environment, learning how to dive and find food [157]. Numerous studies have used pup movement data to address the ontogeny of diving from a physiological perspective [40, 157, 167, 168], and comparatively fewer investigate the ontogeny of foraging strategies [123, 156, 169]. For otariids, and some phocid species, pups may learn to dive (and potentially forage) alongside their mother [16, 38, 39]. However, some phocid species, such as elephant and grey seals, undergo a post-weaning fast, often on land, and must learn to dive and find food without parental supervision before their energy stores are exhausted [170, 171]. Breed et al. [123] modelled movement data for young-of-the-year (YOY; captured at five months of age) and sub-adult grey seals using an SSM and found evidence that sex-related differences in foraging may develop before sexual dimorphism emerges. They also found that YOY animals travelled up to three times further to foraging patches than sub-adults and adults, requiring greater transit time and energetic investment [123]. Given that pups are already constrained in terms of accessible foraging habitat by their limited physiological capacity to dive to, and remain at, depth, this has potentially important ramifications for survival [157, 172]. Pups gain lean mass rather than blubber in their first year of independent feeding [85]. Failure or delay in successful foraging after leaving the natal colony is likely to result in depletion of limited protein, and ultimately starvation [170]. Therefore, smaller pups with more limited fuel reserves may not develop the necessary physiological capability to exploit foraging grounds before their protein stores are diminished [157, 170]. More research is needed to fully understand the challenges facing pups as they leave the colony and learn to find food in a rapidly-changing marine environment, so that important foraging areas can be identified and potential anthropogenic impacts can be assessed and effectively mitigated at this critical life stage. Furthermore, integrating more movement sensors such as accelerometers in tags deployed on pups will allow better classification of movement states from location and dive data. Given that pups have different physiological capabilities and energy requirements to adults, and their behaviour will likely change over time, the assumptions of behavioural modes from adult foraging models may be inaccurate.

## Conclusions

### Concluding remarks

As we continue to impact marine ecosystems with over-fishing; increased vessel traffic; habitat modification; pollution, and anthropogenic climate change, rates of biodiversity loss may pass a critical threshold of extinction [173]. In addition to these pressures, the ramifications for marine fauna of policy changes such as fisheries discard reforms, and the switch from hydrocarbon extraction to marine renewable energy installations, remain unknown. Assessing the significance of these changes for marine ecosystems will be of chief importance for conservation management [173, 174]. Among the species likely to be most immediately and obviously affected are marine predators [19, 130, 175]. Accurately reconstructing predator foraging movements will be crucial to identifying critical habitat for marine species and designing effective Marine Protected Areas (MPAs) that will benefit entire ecosystems [176–179]. Moreover, marine mammals represent a valuable resource as sentinels of ecosystem health, and expanding our knowledge of their foraging behaviour will allow us to assess how marine systems may respond under global environmental change [180–183]. Biologging data will no doubt play a leading role in this process, and further refining analytical techniques of these data should be given high priority [144]. There remain inherent limitations in inferring animal behaviour from location and dive data. No one analytical approach can capture foraging from these data with complete accuracy. However, ecologists can select the best analytical method based upon several key considerations; the research question, the study species, the number and class of individuals required, the device type, and device programming (Fig. 6). Depending on the range of movement of the individual, the resolution of the data and the complexity of the analysis, some techniques may over or under-estimate foraging. Nevertheless, SSMs represent a rapidly-developing holistic statistical method that has the capacity to incorporate multiple data types and allows more robust behavioural inferences to be made [111]. SSMs will allow ecologists to create a more complete picture of activity budgets and population dynamics [121, 122], with the potential to draw links between predator behaviours and environmental phenomena [128]. The priority for future work is to focus on incorporating oceanographic information into analyses to better understand patterns of habitat use, to determine the physical and behavioural consequences of specific tags to the study animal, and to develop an understanding of the ontogeny of foraging strategies in naïve pups. This will lead to more accurate population-level assessment of habitat use and will therefore benefit our ability to mitigate the effects of anthropogenic activity on the marine environment.

## Additional file

Additional file 1: Glossary. (DOCX 13 kb)

## Abbreviations

ADL: Aerobic Dive Limit
ARS: Area Restricted Search
BODC: British Oceanographic Data Centre
CLS: Collecte Localisation Satellites
CTD: Conductivity Temperature Depth
FPT: First-Passage Time
GLS: Global Location Sensor
GPS: Global Positioning System
GSM: Global System for Mobile Communications
HMM: Hidden Markov Model
KF: Kalman Filter
LC: Location Class
LS: Least Squares
MCMC: Markov Chain Monte Carlo
ML: Maximum Likelihood
MPA: Marine Protected Area
NEODAAS: NERC (Natural Environment Research Council) Earth Observation Data Acquisition and Analysis Service
NOAA: National Oceanographic and Atmospheric Administration
ODT: Optimal Dive Theory
OFT: Optimal Foraging Theory
PODAAC: Physical Oceanography Distributed Active Archive Center
RT: Residence Time
SMRU: Sea Mammal Research Unit, University of St Andrews
SPOT: Smart Position or Temperature Transmitting
SRDL: Satellite Relay Data Logger
SSM: State-Space Model
SST: Sea Surface Temperature
STT: Stomach Temperature Telemetry
TAD: Time Allocation at Depth
TDR: Time-Depth Recorder
UHF: Ultra High Frequency
VHF: Very High Frequency
YOY: Young-Of-the-Year
