# Supplementary material for: Navigating uncertain waters: a critical review of inferring foraging behaviour from location and dive data in pinnipeds
Source: Mov Ecol. 2016 Oct 26;4:25. doi: 10.1186/s40462-016-0090-9 (PMC5080796; doi:10.1186/s40462-016-0090-9)
Supplement: Additional file 1: — Glossary. (DOCX 13 kb) [file 40462_2016_90_MOESM1_ESM.docx]

**SUPPLEMENTARY MATERIAL**

**Glossary**

**Aerobic dive limit (ADL):** The point during a dive at which lactate begins to accumulate in the blood. This is either classified as ‘observed’ (measured) ADL or ‘calculated’ ADL (estimating oxygen reserves divided by estimated rate of consumption).

**Area restricted search (ARS):** Foraging pattern characterised by slow movements and high turning rate whereby an animal concentrates its search in a specific small scale area relative to its overall scale of movement. This may be triggered by a recently encountered prey resource or the presence of other foragers (local enhancement).

**Biologging:** Use of animal-attached tags to record (and possibly transmit) data relating to that animal’s movement, behaviour, physiology and/or environment.

**Biotelemetry:** Biologging using devices that autonomously transmit data via satellite or radio telemetry, or mobile phone technology, as opposed to archival loggers.

**Conductivity temperature depth (CTD):** A multi-sensor logger that can record depth as well as water temperature and salinity. These can be incorporated in satellite relay units. A major advantage is that, when deployed on a diving animal, they can record a vertical temperature and salinity profile for the water column as the animal moves through it.

**Displacement:** Track metric calculated as the distance travelled between successive location fixes (Figure 4b). Used in conjunction with other metrics such as turning angle to identify area restricted search (ARS) behaviour. Fixes are often regularised to a constant time step before calculating (Figure 4a).

**First-passage time (FPT):** Track metric calculated as the time taken for an animal to cross a circle of given radius (defined by the maximum peak in FPT relative variance). Used to identify area restricted search (ARS) behaviour (Figure 5b-c).

**Hidden Markov model (HMM):** Form of state-space model (SSM) often used to classify discrete movement states (from which behaviours such as ‘resting’, ‘foraging’ or ‘travelling’ can be inferred) from movement metrics such as turning angle, displacement, and diving activity (Figure 5d).

**Kalman Filter (KF):** Form of state-space model (SSM) often used to improve the location accuracy of Argos tracking data.

**Optimal dive theory (ODT):** Application of optimal foraging theory (OFT) to diving animals, predicting that animals maximise foraging effort within the limitations of oxygen reserves, rarely reaching aerobic dive limit (ADL).

**Optimal foraging theory (OFT):** Theory of animal foraging that predicts the best strategy for maximising energy gain (and therefore fitness) whilst minimising energetic cost by concentrating effort in areas of successful feeding.

**State-space model (SSM):** Time series model that predicts the future state of an object given its previous states in a probabilistic manner. Can be used to improve location accuracy for low-resolution data (e.g. Kalman filter; KF) or to determine allocation of behavioural states along a track (e.g. hidden Markov model; HMM).

**Stomach temperature telemetry (STT):** An animal’s stomach temperature can be recorded by STT devices, which are retained in the animal’s stomach for several days. Sharp drops in temperature are generally accepted to indicate ingestion of cold prey.

**Time allocation at depth (TAD) index:** Analytical method for quantifying the allocation of time throughout the time-depth profile of a dive. Index values close to 1 indicate a dive where the individual has spent longer at the bottom phase of the dive than in ascent and descent (i.e U-shaped dive). Values close to 0.5 indicate an equal distribution of time across all depths (i.e. V shaped dive). Higher values are often used to infer foraging.

**Time-depth recorder (TDR):** Miniaturised sensor recording depth (via pressure) as a function of time. Sensors can be activated by a salt-water switch or pressure/depth threshold.

**Turning angle:** Track metric calculated as the change in bearing from one location fix to the next (Figure 4b). Often used in conjunction with other metrics to identify area restricted search (ARS) behaviour.
